# Assessment of root-specific promoters in banana and tobacco and identification of a banana TIP2 promoter with strong root activity

**DOI:** 10.3389/fpls.2022.1009487

**Published:** 2022-10-05

**Authors:** Anthony James, Jean-Yves Paul, Jacqueline Souvan, Tal Cooper, James Dale, Rob Harding, Pradeep Deo

**Affiliations:** Centre for Agriculture and the Bioeconomy, Queensland University of Technology, Brisbane, QLD, Australia

**Keywords:** aquaporin, banana, β-glucuronidase, root-specific expression, promoter, tonoplast intrinsic protein (TIP)

## Abstract

Genetic modification is one possible strategy to generate bananas (*Musa* spp.) with resistance to the soil-borne pathogen causing Fusarium wilt. The availability of banana root-specific promoters to target transgene expression to the sites of infection would be beneficial. We have assessed 18 promoter sequences derived from a range of plant species for their expression profiles in banana tissues to identify those with root-specific activity. Promoter sequences were isolated and fused to the β-glucuronidase (GUS) gene to assess their expression levels and tissue specificity in both banana and the model plant tobacco. Two heterologous promoters conferring high root expression levels in banana were identified, including a β‐glucosidase 1 (GLU1) promoter from maize and the RB7-type tonoplast intrinsic protein (TIP)-2 promoter from strawberry. Further, a novel Musa TIP2-2 promoter sequence was isolated and characterized which, when fused to the GUS gene, conferred very high GUS expression levels in banana roots. These promoters will expand the options for the control of gene expression in genetically modified bananas, providing a tool to develop plants with resistance not only to soil-borne diseases such as Fusarium wilt, but also for the improvement of other traits, such as nematode resistance, nutrition or abiotic stress resistance.

## Introduction

Banana (*Musa* spp.) is an important commercial and staple food crop. Grown throughout tropical and subtropical regions, banana production is subject to numerous biotic constraints, including serious diseases caused by various fungi, bacteria and viruses (Jones, 2018). One of the most destructive fungal diseases of bananas is Fusarium wilt, caused by the soil-borne fungus *Fusarium oxysporum* f. sp. *cubense* (Foc) (Ploetz, 2015). Foc is comprised of several genetic variants (known as races) which affect specific banana cultivars. Foc race 1 was responsible for the destruction of more than 40,000 ha of Gros Michel bananas in south and central America in the 1950s (Ploetz, 2005). It was only through the replacement of Gros Michel with the resistant Cavendish cultivar that the global export trade was saved. However, the emergence of a new variant of Foc, known as Tropical Race 4 (TR4), in south-east Asia in the 1970s, and its subsequent spread in Asia (Mostert et al., 2017; Zheng et al., 2018), Australia (Pegg et al., 2019), the Middle East (Maymon et al., 2020), Africa (Butler, 2013) and more recently South America (García-Bastidas et al., 2020) poses a threat to the production of Cavendish and numerous other banana cultivars. There are no effective long-term control options for managing Foc, other than exclusion and host plant resistance (Pegg et al., 2019).

Although wild bananas with resistance to Foc have been identified (Li et al., 2015; Dale et al., 2017a; Ahmad et al., 2020), the introgression of desirable traits into commercially preferred Cavendish cultivars through conventional breeding is extremely difficult (Aguilar Morán, 2013; Ploetz, 2015; Dale et al., 2017b). Genetic modification is an alternative approach to improving existing banana cultivars without affecting key farmer- and consumer-preferred traits. Since the development of efficient banana transformation protocols (May et al., 1995; Sági et al., 1995), there have been numerous reports on the development of banana plants with improved nutritional content as well as resistance to a range of biotic and abiotic stresses (Ghag and Ganapathi, 2017; Paul et al., 2017; Pua et al., 2019; Tripathi et al., 2019). In 2017, we reported the generation of several Cavendish banana lines with Foc TR4 resistance following a three-year field trial in a heavily Foc TR4-infested site in the Northern Territory, Australia (Dale et al., 2017a). These resistant lines constitutively expressed either a CC-NBS-LRR-type resistance gene analogue (RGA) isolated from a wild, diploid *M. acuminata* ssp. *malaccensis* (Mam) banana, MamRGA2, or Ced-9, a stress tolerance gene from the nematode worm *Caenorhabditis elegans*. To mitigate any perceived biosafety concerns and therefore improve consumer acceptability, limiting the expression of these transgenes to the roots, which are the sites of Foc infection, would be desirable. The most suitable strategy for restricting gene expression to specific plant tissues is the use of tissue-specific promoters (Kummari et al., 2020).

Root-specific promoters have been reported from a wide range of plants including model species such as *Arabidopsis thaliana* as well as from agriculturally important crop plants. Some examples from *A. thaliana* include the ‘root-specific kinase homolog 1’ (Hwang and Goodman, 1995), the ‘ethylene insensitive root 1’ (Sieberer et al., 2000), and the ‘myrosinase-associated protein pyk10’ (Nitz et al., 2001), while examples of root-specific promoters from dicotyledonous crop plants include ‘proline rich proteins’ from soybean (Suzuki et al., 1993) and alfalfa (Winicov et al., 2004) and several sugar beet taproot-specific promoters, including ‘thaumatin-like protein’, ‘linker histone variant 1’, and ‘major latex-like protein’ (Oltmanns et al., 2006) among many others. Although several root-specific promoters have been characterized in important monocotyledonous crops such as rice and maize (Xu et al., 1995; Gu et al., 2006; Xie et al., 2010), there are no reports characterizing root-specific promoters in banana.

Aquaporins, or water channel proteins, are a group of major-intrinsic proteins involved in transport of water and metabolites across biological membranes (Chaumont et al., 2001; Johanson and Gustavsson, 2002; Kaldenhoff and Fischer, 2006). Plant aquaporins constitute a large family which are divided into four subgroups, namely the tonoplast intrinsic proteins (TIPs), plasma-membrane intrinsic proteins (PIPs), Nod26-like intrinsic proteins (NIPs) and the small-basic intrinsic proteins (SIPs), with more than 30 members identified in the genomes of *A. thaliana* (Johanson and Gustavsson, 2002), maize (Chaumont et al., 2001) and rice (Sakurai et al., 2005). Expression studies on the different aquaporin genes in rice and maize have identified a number of these genes with tissue-specific expression patterns (Chaumont et al., 2001; Sakurai et al., 2005; Sakurai et al., 2008). In several cases, specific aquaporin genes were shown to have high levels of expression in root tissues, or even root-specific expression. In rice, this includes some PIP and TIP-type aquaporins, such as OsPIP2-3/OsPIP2-4/OsPIP2-5 (Sakurai et al., 2005) and OsTIP2-1/OsTIP2-2 (Sakurai et al., 2008), while in maize ZmPIP2-4, ZmTIP2-1 and ZmTIP2-2 showed root-preferred or -specific expression (Chaumont et al., 2001). Further, characterization of the promoters of the ‘RB7-type’ TIPs from tobacco and strawberry also demonstrated their ability to drive root-specific expression in their respective host plants (Yamamoto et al., 1991; Vaughan et al., 2006).

In this study, we assessed the root-specific expression in bananas of a collection of previously characterized plant-derived promoters with highly root-preferred or root-specific activity in other plant species. As regeneration of transformed bananas into plantlets is a lengthy process, in parallel we also transformed the model plant *Nicotiana tabacum* (tobacco) to rapidly assess the functional activity of all the promoters. Further, we analyzed the upstream regions of two *M. acuminata TIP2-2* genes (*MaTIP2-2*) which showed contrasting levels of root-specific expression in banana. The identification of promoters suitable for root-specific transgene expression in bananas will expand the options available in the molecular “toolbox” for research efforts aimed at controlling banana pathogens such as Foc, other pathogens and pests which invade through the roots as well as enhancing tolerance to abiotic stresses such as drought.

## Materials and methods

### Cloning of promoter sequences

Based on a survey of the literature, 16 previously identified root-specific promoters were isolated and assessed in this study (**Table 1**). For each promoter, sequence-specific forward and reverse primers were designed based on the published sequences to amplify either the complete sequence or partial sequences with 5’ truncations (**Table S1**). The forward and reverse primers incorporated *Avr*II and *Asi*SI restriction enzyme sites, respectively, to facilitate downstream cloning.

**Table 1:** List of promoter sequences used for analysis in tobacco and banana.

Promoter name	Classification	Species of origin	GenBank ID	Length of amplified sequence (bp)	% nucleotide similarity to published sequence	Reference
AtRSK1a^1^	Root-specific kinase homolog 1	*Arabidopsis thaliana*	AT2G26290	1197	99.0	Hwang and Goodman, 1995
AtRSK1b				2300	99.0	
AtCrp1	Cryptic root-specific promoter 1		AF242314	2291	99.7	Mollier et al., 2000
AtCrp2	Cryptic root-specific promoter 2		AY601849	460	99.8	Sivanandan et al., 2005
AtEIR1a^1^	Ethylene insensitive root 1		AT5G57090	1218	99.0	Sieberer et al., 2000
AtEIR1b				2157	99.0	
BvMll	Major latex-like protein	*Beta vulgaris*	AX449164	1653	99.1	Oltmanns et al., 2006
BvTlp	Thaumatin-like protein		AM110765	2130	74.5	
FaRB7	Tonoplast intrinsic protein	*Fragaria ananassa*	DQ178022	974	95.0	Vaughan et al., 2006
GmPRP1	Proline rich protein 1	*Glycine max*	J02746	1041	99.4	Suzuki et al., 1993
MaTIP2-2a	Tonoplast intrinsic protein	*Musa acuminata*	Ma11_p04350.1	1483	99.7	This study
MaTIP2-2b			Ma11_p19380.1	802	98.9	
MsPRP2	Proline rich protein 2	*Medicago sativa*	AF028841	649	99.1	Winicov et al., 2004
NtRB7a^1^	Tonoplast intrinsic protein	*Nicotiana tabacum*	S45406	684	99.9	Yamamoto et al., 1991
NtRB7b				1321	99.8	
OsRCg2	Root-specific cDNA	*Oryza sativa*	L27210	1642	99.8	Xu et al., 1995
ZmGLU1	β‐glucosidase 1	*Zea mays*	DQ333310	1720	99.8	Gu et al., 2006
ZmPR10.1	Pathogenesis-related protein 10		AC203883	1495	89.4	Xie et al., 2010

^1^a denotes a 5’ truncated version of the same sequence described in b.

Arabidopsis RSK1 and EIR1 promoter sequences were amplified by PCR from plasmids previously prepared at QUT (Facy, 2009) and were subsequently cloned as described below. The remaining promoters were amplified from total nucleic acid (TNA) extracted from either leaf tissue (banana, tobacco, arabidopsis, maize, rice, alfalfa and sugar beet) or seed (soybean) using either the DNeasy^®^ Plant Mini Kit (Qiagen, Australia), or a cetyltrimethylammonium bromide (CTAB)-based method (James et al., 2011). PCR amplification was carried out using either GoTaq^®^ Green Master Mix (Promega, USA) or Expand Hi Fidelity DNA polymerase (Roche, Australia) with 10 ng TNA as template and 10 ρmol of primers in a final volume of 20 μL. PCR cycling conditions were 94°C initial denaturation for 2 min followed by 35 cycles of 94°C denaturation for 20 s, 50°C annealing for 20 s, 68/72°C extension for Expand/GoTaq respectively for 1 min per kb and a final extension step at 72°C for 2 min. PCRs were visualized on agarose gels and amplicons gel-purified using the Freeze ‘N Squeeze DNA Gel Extraction Spin Columns (Bio-Rad, USA), ligated into pGEM^®^-T Easy (Promega, USA) and transformed into competent *Escherichia coli* XL-1 Blue cells by heat-shock. Putative recombinant clones were identified by blue/white selection and plasmid DNA was purified by alkaline lysis (Sambrook and Russell, 2001). Inserts were confirmed by restriction with *Not*I and DNA sequencing was subsequently carried out using BigDye™ Terminator v3.1 Cycle Sequencing Kit (Applied Biosystems, Australia). Sequences were analyzed using Vector NTI Advance^®^ 11 software (Thermo Fisher Scientific, Australia).

The promoters associated with two putative banana TIP2-2 sequences were also isolated from purified banana DNA (*M. acuminata* ssp. *malaccensis*) and their root specificity assessed (**Table 1**). Musa TIP sequences were identified from the Banana Genome hub (https://banana-genome-hub.southgreen.fr/) using a combination of Blast and keyword searches into the *M. acuminata* ‘DH-Pahang’ (version 2) genome database (**Table S2**). Retrieved TIP sequences were aligned together with rice (Sakurai et al., 2005) and maize (Chaumont et al., 2001) TIP sequences, as well as tobacco RB7 (NtRB7) (Yamamoto et al., 1991), strawberry RB7 (FaRB7) (Vaughan et al., 2006) and tomato RB7 (SlRB7) (GenBank accession no. AAB53329). Sequences were aligned in MEGA7 (Kumar et al., 2016) and phylogenetic reconstruction carried out using the Neighbor-Joining method with bootstrap analysis (1000 replications; **Figure S1**). Nucleic acid sequences from the region upstream of two genes (Ma11_p04350.1 and Ma11_p19380.1) were subsequently downloaded from the ‘DH-Pahang’ genome database and specific primers designed (**Table S1**) to amplify up to approx. 1.5 kb of upstream region from the first ATG initiation codon in the annotated sequences. PCR, cloning and sequencing was carried out as described previously.

### Preparation of promoter-*uidA* constructs

The binary plasmid pYC34 containing the *A. thaliana* Bcl-2 associated athanogene 4 (AtBAG4) promoter driving the expression of the *uidA* gene (encoding the enzyme β-glucuronidase, derived from pCambia1305) was used as a reference vector for cloning. The AtBAG4 promoter sequence was flanked by 5’ *Avr*II and a 3’ *Asi*SI restriction enzyme sites, allowing the promoter sequences to be conveniently exchanged. Therefore, all promoter fragments described previously were excised from pGEM^®^-T Easy clones by restriction enzyme digestion using *Avr*II/*Asi*SI and subsequently ligated into pYC34 previously digested with the same enzymes. All constructs were sequenced to confirm the presence, orientation and integrity of the respective promoter fragments.

### Generation and maintenance of transgenic plants

The promoter-*uidA* constructs were introduced into *Agrobacterium tumefaciens* strain LBA4404 or AGL-1 by electroporation, for tobacco and banana transformation, respectively. Single colonies were identified and cultured for 48 h in LB media supplemented with 100 mg/L spectinomycin and 50 mg/L rifampicin. Each culture (1 μL) was screened by PCR for the presence of the binary vector using GoTaq Green, a promoter-specific forward primer and the Gus-R *uidA*-specific reverse primer (**Table S3**). Tobacco (*Nicotiana tabacum* cv. Samsun) plants were maintained on MS media with monthly subculturing. Leaf disc transformation was carried out as described by Horsch et al. (1989) and, to ensure that all transgenic tobacco lines generated were independent of each other, a single line was regenerated from each leaf disc. Regenerating plantlets were cultured on MS media supplemented with 100 mg/L kanamycin to select transformants and 200 mg/L timentin to control residual Agrobacterium. Banana (*Musa* spp. cv. Lady Finger; AAB subgroup) embryogenic cell suspensions (ECS) were initiated, maintained and transformed as described by Khanna et al. (2004). Transformed banana embryogenic cells were maintained on successive tissue culture media in 90 mm tissue culture plates. To ensure that every regenerated transgenic banana line originated from a unique transformation event, only one single line per 90 mm plate was regenerated on MS media supplemented with kanamycin and timentin as described above. Putative transgenic tobacco and banana lines were then grown in a plant growth chamber at 23 and 27°C, respectively, under fluorescent lights with a 16 h photoperiod.

### Transgenic plant identification

Leaf samples were collected from all putative transgenic plants *in vitro* and TNA isolated using the CTAB-based method described previously. The presence of the respective promoter-*uidA* cassette was confirmed in each plant by PCR using a promoter sequence-specific forward primer and primer Gus-R (**Table S3**) using GoTaq Green and as described previously. Confirmed transgenic tobacco plants were multiplied from nodal cuttings while confirmed transgenic banana plants were multiplied as described by Khanna et al. (2004).

### Reporter gene assays and detection of β-glucuronidase (GUS) activity

GUS expression was visualized histochemically while GUS activity was assayed fluorometrically essentially as described by Jefferson et al. (1987). For tobacco, following multiplication, a maximum of five lines selected from independent leaf discs were analyzed. One plant per line was histochemically stained while three plants per line were sampled for fluorometric assays. For banana, a maximum of eight independent lines were analyzed with a single plant per line stained histochemically and a single plant per line sampled for fluorometric analysis.

For histochemical staining, whole tobacco plants were removed from tissue culture flasks, the roots rinsed free of media using tap water and plants vacuum infiltrated for 30 min in a solution of 50 mM Na_3_PO_4_, 0.01 M EDTA, 500 nM ferrocyanide, 500 nM ferricyanide, 0.1% (v/v) Triton X-100 and 1 mM 5-bromo-4-chloro-3-indolyl-beta-D-glucuronic acid (X-Gluc) (PhytoTechnology Laboratories, USA) and incubated at 37°C for 24 h. Following staining, chlorophyll was removed by immersion in ethanol. Banana plantlets were treated similarly, however, the staining solution was modified to contain 100 mM tri-sodium citrate (El-kereamy et al., 2012) and de-staining was carried out using ethanol:acetic acid (50:50 v/v). Plants were then stored in 100% ethanol and photographed using a EOS 750D Digital-SLR camera (Canon, Japan).

Fluorometric assays were carried out using leaf and root tissue protein extracts as described by Jefferson et al. (1987). Plants were removed from culture vessels and media rinsed from roots using tap water. Excess water was removed by blotting on paper towel and the roots detached from the base of the plant and placed into 5 mL tubes with one 5 mm lead bead. Leaf tissue was removed from the base of the petiole and placed into a separate tube. Samples were immediately frozen in liquid nitrogen and either processed directly (banana) or placed into a -80°C freezer overnight and subsequently lyophilized for 24 h. Frozen/lyophilized leaf and root samples were powdered using a Mini-Beadbeater-8 (Biospecproducts, USA). As a comparison for promoter activity in banana, three plants transformed with pUbi-*uidA* (containing a Maize poly-ubiquitin promoter sequence upstream of *uidA*) were kindly provided by Dr Cara Mortimer (QUT).

Total soluble protein (TSP) was extracted for each sample as per Jefferson et al. (1987) and quantified undiluted using Bradford assays (Bradford, 1976). For fluorometric assays, extracts were diluted between 1/10 and 1/80 to ensure that each reading fitted within the range of the associated standard curve. Three biological replicates were assessed for tobacco (each in triplicate), while for banana a single biological replicate was assessed in triplicate. Fluorescence was measured using a LS50B luminescence spectrophotometer (PerkinElmer, USA).

### Statistical analysis

D’agostino-Pearson and Levene’s tests were used to test for normality and homogeneity of variances, respectively. Independent samples were analyzed with the non-parametric Kruskal-Wallis test and multiple comparisons made with the Dunn’s *Post-hoc* test with p-values adjusted using the Benjamini-Hochberg method. The analyses were undertaken in R using base statistical functions and the packages fBasics v3011.87, car v3.0-10, rstatix v0.6.0 and PMCRplus v.1.7.1.

## Results

### Amplification and cloning of promoter sequences

A total of 18 candidate promoters isolated from nine plant species were characterised in this study (**Table 1**). Sixteen of these promoter candidates were chosen because of their previously reported root specificity while the remaining two were promoters associated with putative banana TIP2-2 genes. For the 16 known promoters, PCR primers were designed from published sequences to specifically amplify each promoter from their respective host plant species (**Table 1**). In three cases (NtRB7, AtEIR1 and AtRSK1) primers were designed to amplify two promoter fragments of different lengths. For 15 out of 16 published sequences, amplicons of the expected size were obtained. However, for the FaRB7 promoter region, a promoter fragment of only 974 bp could be amplified despite attempts to amplify several larger fragments. When the nucleotide sequence of the amplified fragments was compared with their published counterparts, identities of 95% or greater were confirmed for 14 of the promoter sequences, while the ZmPR10.1 and BvTlp promoters showed only 89.4% and 74.5% nucleotide identity, respectively (**Table 1**).

Based on previous reports describing the root-specific expression of *TIP2* genes in maize and rice, attempts were made to identify and characterise homologous genes in banana. Based on a keyword search in the annotated *Musa* genome sequence, a collection of putative *Musa* aquaporin genes was identified. The keyword search returned 50 matches under the description of ‘aquaporin’ or ‘probable aquaporin’, including 21 PIPs, 17 TIPs, nine NIPs and three SIPs (**Table S2**). TIP protein sequences from rice, maize and banana (together with characterised RB7-type TIP2 sequences from tobacco, tomato and strawberry) were aligned using MEGA7 and a phylogenetic tree constructed (**Figure S1**). Two putative *MaTIP2-2* gene sequences designated *MaTIP2-2a* (Ma11_p04350.1) and *MaTIP2-2b* (Ma11_p19380.1) were selected for further analysis based on their clustering with the RB7 sequences and TIP2 sequences of maize and rice. The upstream sequences of these genes were subsequently amplified, cloned and sequenced. For the MaTIP2-2a promoter sequence, a 1483 bp region was cloned and sequenced which showed 99.7% identity to the published DH-Pahang genomic sequence at the nucleotide (nt) level. For the MaTIP2-2b promoter sequence, a 802 bp region was amplified with 98.9% identity to the published DH-Pahang genomic sequence. Attempts to amplify a longer fragment of this promoter were unsuccessful.

In total, 18 promoter sequences were amplified, cloned and sequenced (**Table 1**). Each promoter sequence was subsequently sub-cloned into the plant binary expression vector, pYC34, upstream of the *uidA* reporter gene prior to transformation into tobacco and banana.

### Analysis of transgenic tobacco lines

To assess their functionality, all 18 promoter constructs were transformed into tobacco in three separate experimental groups. Plants were regenerated, sampled and analysed in these three groups. For each promoter construct, putatively transformed plants were regenerated and the presence of the promoter-*uidA* cassettes confirmed in each plant by PCR (results not shown).

**Figure 1:**
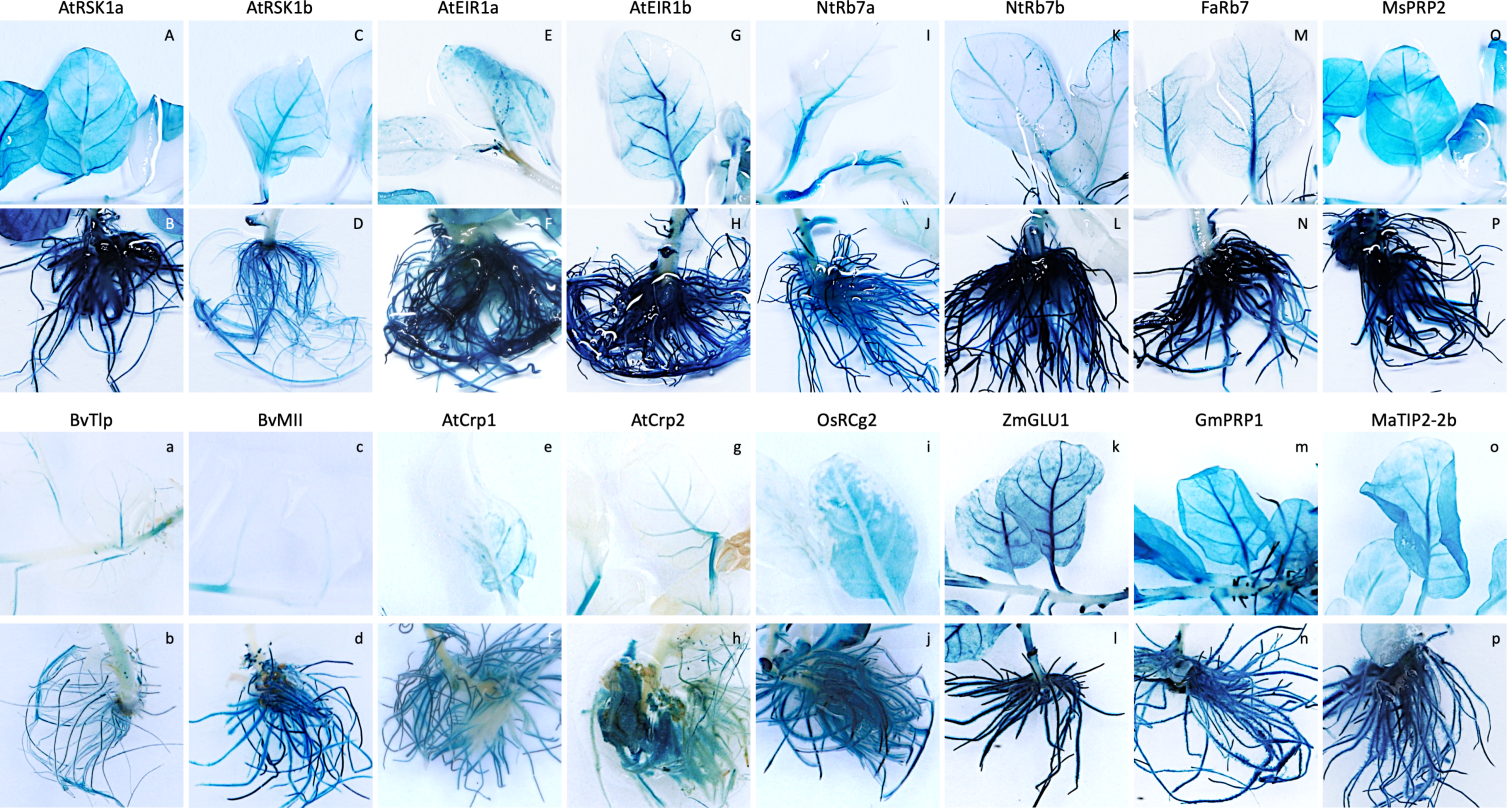
Expression pattern of promoter-*uidA* constructs in leaf and root tissues of selected transgenic tobacco lines. Transgenic promoter-*uidA* tobacco plants were generated and whole plants were stained to visualize GUS expression. Promoter constructs shown include **(A, B)** *Arabidopsis thaliana* root-specific kinase homolog 1a (AtRSK1a), **(C, D)** AtRSK1b, **(E, F)** *A thaliana* ethylene insensitive root 1a (AtEIR1a), **(G, H)** AtEIR1b, **(I, J)** *Nicotiana tobacum* Rb7a NtRb7a, **(K, L)** NtRb7b, **(M, N)** *Fragaria ananassa* Rb7 (FaRb7), **(O, P)** *Medicago sativa* proline rich protein 2 (MsPRP2), **(A, B)** *Beta vulgaris* thaumatin-like protein (BvTlp), **(C, D)** *B vulgaris* major latex-like protein (BvMll), **(E, F)** *A thaliana* cryptic root-specific promoter 1 (AtCrp1), **(G, H)** AtCrp2, **(I, J)** *Oryza sativa* root-specific cDNA 2 (OsRCg2), **(K, L)** *Zea mays* β-glucosidase 1 (ZmGLU1), **(M, N)** *Glycine max* proline-rich protein 1 (GmPRP1) and **(O, P)** *Musa acuminata* tonoplast intrinsic protein 2-2b (MaTIP2-2b).

In the first transformation experiment, the activity of eight of the promoter sequences (two AtRSK1, two AtEIR1, two NtRB7, FaRB7 and MsPRP2) was assessed (**Table 2**). For each promoter construct, five individual lines were regenerated, except for the AtRSK1a and AtEIR1b constructs for which only four and three lines were regenerated, respectively. Initially, promoter activity was evaluated by histochemical staining of whole plants followed by a visual assessment (**Figure 1**; **Table 2**). Of the nine plants transformed with the AtRSK1a/b promoter constructs, 3/4 (75%) and 5/5 (100%), respectively, showed strong GUS expression in the roots (**Table 2**). Visible GUS expression in the stem and leaf lamina of these plants was weak to moderate and was only observed in 50 and 20% of the respective plants, whereas expression in the leaf vasculature was observed in 25 and 80% of the respective plants and was considered moderate to high (**Figures 1A-D**, **S2A1-B3**). In plants transformed with the AtEIR1a/b constructs, 4/5 (80%) and 3/3 (100%) plants respectively showed strong GUS expression in the roots, while expression in the stem and leaf vasculature tissues was only weak to moderate in 5 plants and high in one AtEIR1b plant (**Figures 1E-H**, **S2 C1-D3**). One plant each of AtEIR1a and b also showed strong visible GUS expression in the leaf lamina (**Figure S2, C3, D3**). All ten tobacco plants transformed with either of the NtRB7a/b constructs had consistently high root GUS expression. While the intensity of expression remained weak in all other tissue type of these plants, it occurred more often in the stem (60% of the plants) and the leaf vasculature of the NtRB7b plants (100%) (**Figures 1I-L**, **S2 E1-F3**). Four out of five tobacco plants transformed with the FaRB7 promoter showed moderate to strong visible GUS expression in the roots, although the roots of one plant showed no staining (**Figure S2, G1**). Interestingly, the overall expression pattern appeared similar to that obtained from the NtRB7a promoter, with a more consistent moderate expression of GUS observed in the vascular tissues of the petioles and leaves (**Figures 1M-N**, **S2 G1-3**). All five plants transformed with the MsPRP2 promoter showed visible GUS expression in all tissue types which was consistently strongest in the roots followed by the leaf lamina, vasculature and stem (**Table 2**, **Figures 1O, P**, **S2 H1-3**).

**Table 2:** Summary of histochemical GUS staining results for all transgenic tobacco plants.

Promoter name	Experiment	Number of lines	Visual assessment of GUS expression (% of plants and intensity)			
			Root	Stem	Leaf vasculature	Leaf lamina
AtRSK1a^1^	1	4	75% +++	50% +	25% +++	25% +++
AtRSK1b	1	5	100% ++, +++	20% ++	80% ++, +++	20% ++
AtCrp1	2 & 3	5	100% +, ++, +++	60% +	80% +	20% +
AtCrp2	2 & 3	5	80% +, ++	40% +	80% +	0%
AtEIR1a^1^	1	5	80% +++	40% +, ++	20% ++	20% +++
AtEIR1b	1	3	100% +++	34% ++	67% ++, +++	34% +++
BvMll	2 & 3	5	100% +, ++	40% +	0%	0%
BvTlp	2 & 3	4	100% +, ++	50% +	75% +	0%
FaRB7	1	5	80% ++, +++	80% ++	60% ++	20% +
GmPRP1	2 & 3	5	100% ++, +++	100% +	80% +, +++	80% +, +++
MaTIP2-2a^1^	2 & 3	1	0%	0%	100% +	100% +
MaTIP2-2b	2 & 3	5	80% ++, +++	0%	60% +	20% +++
MsPRP2	1	5	100% +++	100% +	100% +, +++	100% +, ++, +++
NtRB7a	1	5	100% +++	60% ++	20% ++	20% +
NtRB7b	1	5	100% +++	60% +	100% +	20% +
OsRCg2	2 & 3	4	100% +, +++	0%	25% +	25% ++
ZmGLU1	2 & 3	5	100% +++	100% +	100% +, +++	60% ++, +++
ZmPR10.1	2 & 3	5	0%	0%	0%	0%

^1^a denotes a 5’ truncated version of the same sequence described in b. GUS expression intensity observed: +, weak; ++, moderate and +++, high.

The second and third experiments assessed the activity of 10 additional constructs (**Table 2**), which included eight promoters derived from arabidopsis (AtCrp1 & 2), maize (ZmGLU1 & PR10.1), soybean (GmPRP1), rice (OsRCg2) and sugar beet (BvMll & Tlp), with the remaining two derived from banana (MaTIP2-2a and 2b). Five independent lines transformed with either the AtCrp1, AtCrp2, ZmGLU1, ZmPR10.1, GmPRP1, BvMll, or MaTIP2-2b promoters were assessed, while only the one available line containing the MaTIP2-2a and four lines containing the BvTlp or OsRCg2 promoters were assessed, respectively. Histochemical staining of whole plants revealed GUS expression in various tissues of all plants except those transformed with the maize PR10.1 promoter for which no visual evidence of GUS expression was observed (**Figures 1a-p**, **S2I1-P3**). Generally, plants transformed with the BvTlp and BvMII promoters had very low levels of GUS expression (**Figures 1a-d**, **S2I1-J3**), with none of the nine plants analysed showing any leaf expression (**Table 2**). Similarly, the AtCrp1 and AtCrp2 promoters generally exhibited weak GUS activity throughout the plants (**Figures 1e-h**, **S2K1-L3**) with only one AtCrp1 plant expressing GUS in the leaves (**Table 2**). GUS expression from the rice RCg2 promoter was observed in the roots of all four plants, but with intensity varying from weak to strong. Further, whereas 1/4 plants showed moderate leaf expression, no plants showed expression in their stem (**Figures 1i-j**, **S2M1-M3**). With minor exceptions, GUS expression in lines transformed with the ZmGLU1 and GmPRP1 promoters was consistently strong in root tissue but weak in the stems (**Table 2**). All but one plant had leaf vasculature expression ranging from weak to strong, while more than 60% of plants expressed GUS in the leaf lamina (**Figures 1k-n**, **S2N1-O3**). Of the two banana TIP2-2 promoters assessed, the activity of MaTIP2-2a was exclusively limited to the leaves of the one plant available for staining (**Figure S2, P1**). In contrast, the MaTIP2-2b promoter was active in almost all tissues (except for stem tissue) but was strongest in roots (**Figures 1o-p**, **S2P2-P3**).

**Figure 2:**
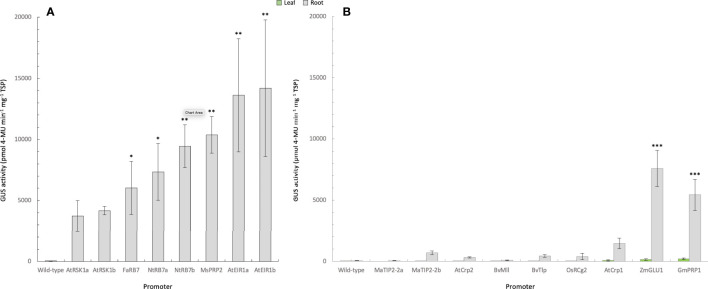
Quantification of GUS activity in leaf and root samples from promoter-*uidA* transgenic tobacco plants. **(A)** GUS activity was measured in the roots (Experiment 1) or **(B)** roots and leaves (Experiment 2 and 3) of transgenic promoter-uidA tobacco plants and reported as mean ρmol 4-methylumbelliferone (MU) min^-1^ mg^-1^ of total soluble protein (TSP) from n biological replicates assessed in triplicate where n was as follows: Experiment 1; wild-type (n=5), Arabidopsis thaliana root-specific kinase homolog 1a (AtRSK1a, n=4) and 1b (AtRSK1b, n=5), Fragaria ananassa Rb7 (FaRB7, n=4), Nicotiana tobacum Rb7a (NtRB7a, n=5) and b (NtRB7b, n=5), Medicago sativa proline rich protein 2 (MsPRP2, n=5), **(A)** thaliana ethylene insensitive root 1a (AtEIR1a, n=4) and 1b (AtEIR1b, n=3). Experiment 2; included wild-type (n=5), Musa acuminata tonoplast intrinsic protein 2a (MaTIP2-2a, n=3) and 2b (MaTIP2-2b, n=15), Beta vulgaris major latex-like protein (BvMll, n=14), **(A)** thaliana cryptic root-specific promoter 1 (AtCrp1, n=14) and 2 (AtCrp2, n=15), Oryza sativa root-specific cDNA 2 (OsRCg2, n=11), **(B)** vulgaris thaumatin-like protein (BvTlp, n=12), Glycine max proline-rich protein 1 (GmPRP1, n=14) and Zea mays β-glucosidase 1 (ZmGLU1, n=14). Non-parametric Kruskal-Wallis test and multiple comparisons with the Dunn’s *Post-hoc* test with p-values adjusted using the Benjamini-Hochberg method. Statistical difference with the wild-type asserted at *<0.05, **<0.01, ***<0.001.

Fluorometric assays were subsequently used to quantitatively assess the level of GUS activity in all transgenic tobacco lines. Only root samples were available from the eight promoters assessed in experiment one. The results from these analyses were generally consistent with those of the histochemical analysis (**Figure 2A**; **Table 2**), and confirmed that all eight promoters were active in tobacco roots. The strongest root GUS activity recorded from the tobacco experiments was from the AtEIR1b promoter with an average expression of more than 14,000 ρmol 4-MU min^-1^ mg^-1^ TSP followed by promoter AtEIR1a, MsPRP2 and NtRB7b (**Figure 2A**). GUS expression in the NtRB7a and FaRB7 lines was significantly lower than these, while the lowest GUS activity levels were measured in the AtRSK1a and b tobacco lines (**Figure 2A**). In experiments 2 and 3, fluorometric assays were carried out on both leaf and root samples from plants transformed with nine different promoters. The results were also consistent with the histochemical assessments (**Figure 2B**). Overall, GUS activity in leaves from all transformed plants was low and only marginally higher than the GUS activity in the wild-type controls. When root samples were analysed, GUS activity was again generally low, with the exception of plants transformed with the ZmGLU1 and GmPRP1 promoters which showed significantly (*p*<0.001) higher activity than the wild-type controls (**Figure 2B**).

### Analysis of transgenic banana lines

Following their assessment in tobacco, the activity of 15 of the promoter sequences was assessed in banana plants. The AtRSK1b and AtEIR1b were omitted because both the full-length and truncated sequences exhibited similar activity in tobacco, while the ZmPR10.1 promoter was omitted due to lack of GUS expression in tobacco. Following transformation of banana ECS, plants were regenerated and tested for the presence of the promoter-*uidA* constructs using PCR as described previously. For each construct, eight PCR-positive independent lines were multiplied in tissue culture until two plant replicates were obtained for each line. One of these plants was selected randomly for histochemical staining, while the remaining plant was analysed by fluorometric assays. Three lines transformed with the maize poly-ubiquitin promoter (ZmUbi) were used as controls.

**Figure 3:**
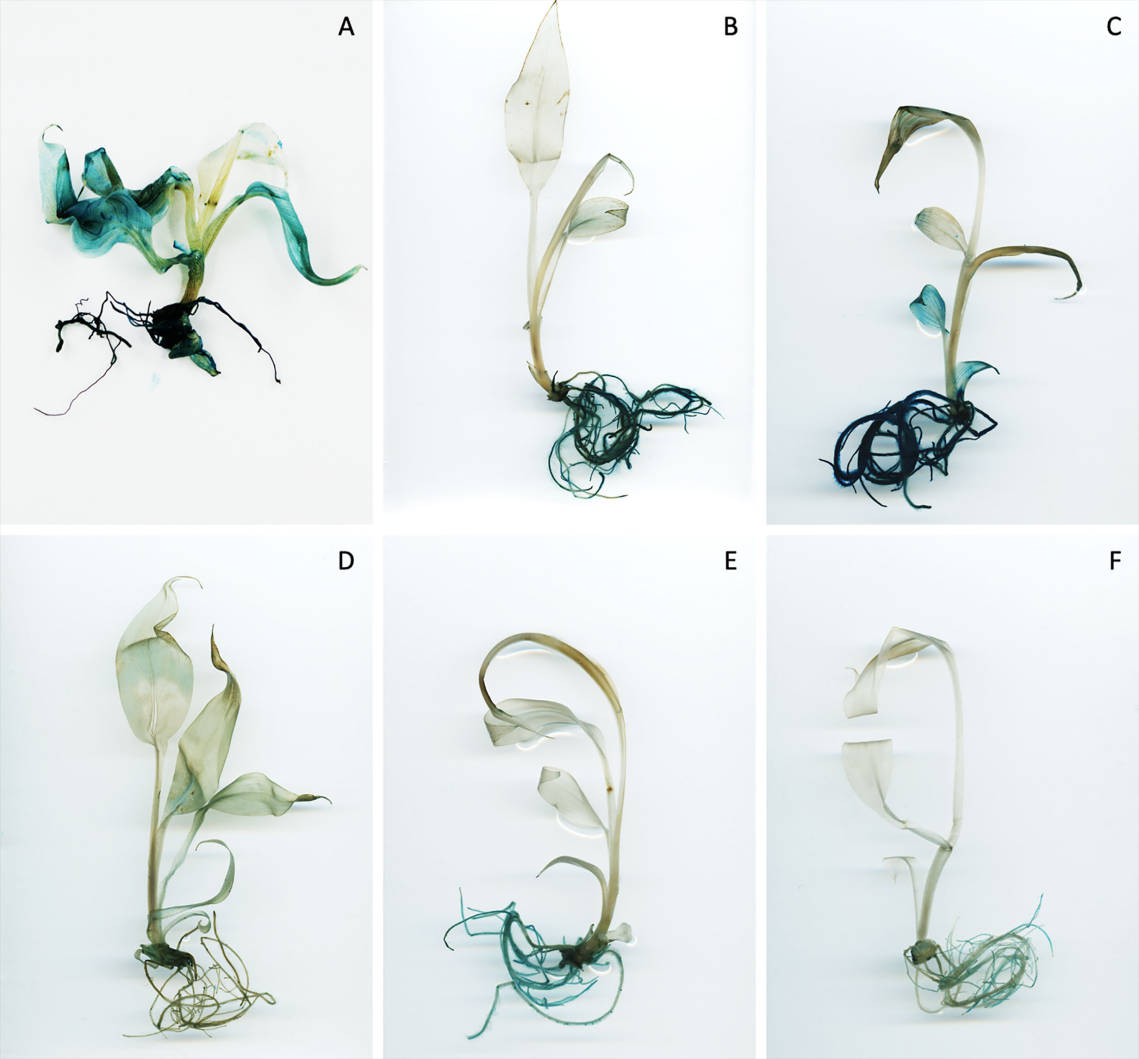
Expression pattern of promoter-*uidA* constructs in root and leaf tissues of selected transgenic banana lines. Transgenic promoter-*uidA* banana plants were generated and whole plants were stained to visualise GUS activity. Representative images selected from eight replicates per construct. Promoter constructs shown include **(A)** ZmUbi, **(B)** FaRb7, **(C)** ZmGLU1, **(D)** OsRCg2, **(E)** MaTIP2-2a and **(F)** MaTIP2-2b.

Histochemical staining of whole banana plantlets revealed that only five of the 15 promoters were active in banana tissue, including three heterologous promoters (FaRB7, ZmGLU1 and OsRCg2) and the two banana TIP2-2 sequences. However, in many instances, the high phenolic content in banana tissues interfered with the staining/de-staining process and hindered analysis. Nevertheless, ZmUbi-*uidA* positive control plants showed strong GUS expression in both leaf and root tissues, with strongest expression in the leaves (**Figure 3A**). Both the FaRB7 and ZmGLU1 promoters showed similarly strong levels of GUS expression, which was almost exclusively restricted to root tissue, while the OsRCg2 promoter showed only low activity, again, predominantly restricted to the roots (**Figures 3B–D**). Of the two *Musa* TIP2-2 promoter sequences assessed, MaTIP2-2a had the highest visible activity of all promoters assessed, and, in most cases, this expression was highly specific to root tissues (**Figure 3E**). In contrast, MaTIP2-2b directed lower levels of GUS expression in banana which was also mainly restricted to the roots (**Figure 3F**).

**Figure 4:**
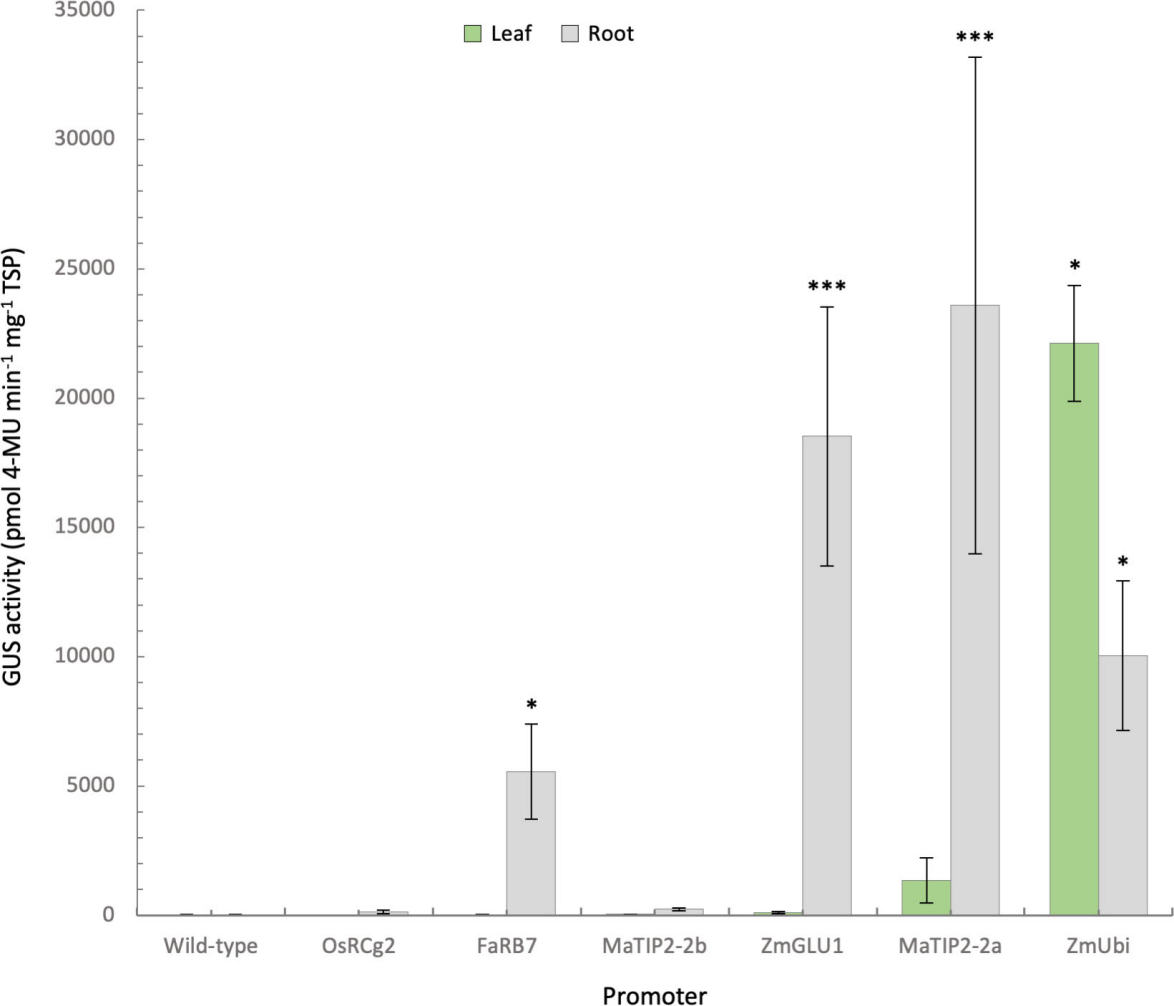
Quantification of GUS activity in leaf and root samples from promoter-*uidA* transgenic banana plants. GUS activity was measured in the leaves and roots of transgenic promoter-*uidA* banana plants and reported as mean ρmol 4-methylumbelliferone (MU) min^-1^ mg^-1^ of total soluble protein (TSP) from 8 biological replicates assessed in triplicate except for wild-type (n=5) and *Zea mays* poly-ubiquitin (ZmUbi, n=3) plants. Non-parametric Kruskal-Wallis test and multiple comparisons with the Dunn’s *Post-hoc* test with p-values adjusted using the Benjamini-Hochberg method. Statistical difference with the wild-type asserted at *<0.05 and ***<0.001.

When GUS protein activity was assessed in each plant using fluorometric assays, the leaves of the ZmUbi control lines had an average GUS activity of approx. 22,000 ρmol 4-MU min^-1^ mg^-1^ TSP, while GUS activity was consistently lower in root tissues of all three lines tested at 10,035 ρmol 4-MU min^-1^ mg^-1^ TSP (**Figure 4**). The strongest GUS activity was measured in the roots of plants with the MaTIP2-2a promoter, with an average of 23,586 ρmol 4-MU min^-1^ mg^-1^ TSP (**Figure 4**). While six of the eight lines transformed with the MaTIP2-2a promoter sequence showed root GUS activity comparable to, or lower than the ZmUbi controls, the other two lines (lines 6 and 7) recorded significantly higher average fluorometric values ranging from 64,267 to 70,499 ρmol 4-MU min^-1^ mg^-1^ TSP. These two lines showed a concomitant higher level of leaf GUS activity compared to the other lines transformed with this promoter, but these levels remained significantly lower than the GUS activity recorded from the leaves of ZmUbi lines (**Figure 4**).

The ZmGLU1 promoter averaged 18,532 ρmol 4-MU min^-1^ mg^-1^ TSP in root tissue (**Figure 4**). In the eight ZmGLU1-*uidA* lines assessed, leaf GUS activity ranged from 10 to 314 ρmol 4-MU min^-1^ mg^-1^ protein, again significantly lower than the average of the ZmUbi lines. This low activity is consistent with the observation of GUS expression restricted to vascular bundles observed in the histochemical staining (**Figure 3**). In contrast, in five of the eight ZmGLU1 lines tested, root GUS activity was equivalent to, or higher than, that of the ZmUbi lines, ranging from 15,410 to 42,188 ρmol 4-MU min^-1^ mg^-1^ protein (**Figure 4**).

In the eight lines transformed with the FaRB7 promoter, root GUS activity averaged 5,550 ρmol 4-MU min^-1^ mg^-1^ TSP, which was statistically higher than the wild-type, but varied from very low in line 7 (146 ρmol 4-MU min^-1^ mg^-1^ TSP) to very high in line 8 (16,091 ρmol 4-MU min^-1^ mg^-1^ TSP). In contrast, leaf GUS activity in all FaRB7 lines was equivalent to wild-type plants. Leaf and root expression measured in the OsRCg2 and MaTIP2-2b lines was not significantly different to wild-type and was consistent with observations from histochemical analyses.

## Discussion

This study aimed to assess a suite of published root-specific promoters to identify a promoter that directed high, root-specific expression in banana. Additionally, two potential root-specific promoter sequences from banana were also identified, isolated and assessed for potential use in the generation of cisgenic banana plants. As the generation of transgenic bananas is a lengthy process taking over 9 months, we initially assessed the functionality of the promoter sequences in tobacco, a model plant which can be rapidly regenerated. Although promoter activity may differ between plant species, especially between monocot and dicot plants (Schäffner and Sheen, 1991; Shimamoto, 1994; Bhattacharyya et al., 2002; Kummari et al., 2020), this approach was considered useful as a rapid screen for promoter function, particularly since many of the isolated promoters were derived from dicot plant species.

In total, 18 promoter-*uidA* constructs were transformed into tobacco and plants regenerated for analysis. Of these, only ZmPR10.1 showed no visible GUS expression in any plants. This inactivity may be explained by the approximately 10% sequence variability between the ZmPR10.1 sequence amplified in the current study and the published sequence, which included a 30 nt insertion and several small deletions (17, 5 and 14 nt). These small changes might affect *cis*-elements necessary for promoter function, or alternatively, transcription factors present in the original host may be absent in tobacco. Histochemical and fluorometric analysis of tobacco plants transformed with the 17 remaining promoter constructs revealed varying GUS expression levels and tissue specificities in tobacco plants (**Figure 1**, **2**, **S2**). Following this analysis, we selected 15 constructs for assessment in banana. Five of the 15 promoters were active in bananas. Of these, the OsRCg2 and MaTIP2-2b promoters directed very low average GUS expression levels in bananas, which, although confined to root tissues, were not significantly higher than wild-type controls. In contrast, the remaining three promoters (FaRB7, ZmGLU1, MaTIP2-2a) directed significant GUS expression levels in banana plants (**Figures 3**, **4**).

Despite the FaRB7 promoter sequence assessed in this study (974 nt) being considerably shorter than the 2,843 nt sequence previously used in strawberry and tobacco (Vaughan et al., 2006), it was still functional in both tobacco and banana. In tobacco, activity was predominantly observed in root tissues, with low expression in the leaf vasculature (**Figures 1M, N**, **S1G1-3**). Similarly, in bananas, FaRB7 was active in root tissues which showed significantly higher root GUS activity than the untransformed control (**Figures 3B**, **4**). However, the average GUS activity of 5,550 ρmol 4-MU min^-1^ mg^-1^ TSP in roots of FaRB7-transformed banana was considerably lower than that reported from strawberry (37,000 ρmol 4-MU min^-1^ mg^-1^ TSP) (Vaughan et al., 2006). Interestingly, in both tobacco and banana, relatively similar levels of root expression were measured in the present study, whereas previously, lower levels of expression were observed in tobacco (Vaughan et al., 2006). These differences may be due to integration site in the plant genome, age of plants used in the different studies, the difference in the length of the promoter sequences, or due to sequence variability between the two promoters, as the sequence we isolated had only 95% nt identity to the published sequence. FaRB7 was the only promoter assessed from a dicot plant species found to be functional in the monocot banana. The extremely low visible expression from the FaRB7 promoter in non-root tissues in banana suggest that it could be useful for root-specific transgene expression in this crop.

The ZmGLU1 promoter sequence we assessed showed an average root GUS activity ~25 times higher than in the leaves of tobacco (**Figure 2B**), which was considerably higher than reported previously. Interestingly, root expression was restricted to the elongation zone, but absent from the region of the root tip, as also described by Gu et al. (2006). This was not the case in banana, however, where GUS activity occurred in all areas of the root. Although GUS staining was also observed in banana leaf vascular tissues, the level of expression in banana leaves was very low compared to the root expression levels (**Figures 3**, **4**). While the leaf expression levels of ZmGLU1 in tobacco and banana were consistent, root expression was significantly higher in bananas than tobacco. The average root GUS expression levels in banana were also significantly higher than the ZmUbi promoter commonly used for transgene expression in monocot plants confirming that, in banana, the ZmGLU1 promoter drives high levels of transgene expression and is highly root preferred.

Since several plant aquaporins, including those encoded by the TIP2-type RB7 genes in tobacco and strawberry (Yamamoto et al., 1991; Vaughan et al., 2006), are known to be root specific, we isolated and characterised the potential promoters associated with two banana TIP2-2 genes. The two putative *Musa* TIP2-2 promoter sequences were identified by sequence similarity with maize and rice TIP2 sequences and their upstream regions were cloned and activity characterised. Both sequences were found to be functional in transgenic tobacco and banana lines, with varying activity. In tobacco, both promoters showed very low leaf and root expression levels which were not significantly different to wild-type (**Figure 2B**). In banana, the MaTIP2-2a promoter showed average GUS expression levels more than double those of the ZmUbi promoter, while average leaf expression levels were comparatively low (**Figure 4**). In two lines, very high GUS expression levels were observed, approximately seven times higher than the ZmUbi promoter, although leaf expression was also considerably higher than wild-type in these two lines. In contrast the remaining six lines showed root expression between 6,047 and 12,994 ρmol 4-MU min^-1^ mg^-1^ TSP, with only one line demonstrating leaf expression higher than the average of wild-type plants. In comparison, the Musa TIP2-2b promoter showed very low levels of root GUS expression in banana, averaging just 233 ρmol 4-MU min^-1^ mg^-1^ TSP and leaf expression equivalent to wild-type plants (**Figure 4**). These results suggest that these two promoters can be used to drive either very high or very low levels of transgene expression in banana roots.

Expression levels can have a critical impact on phenotypes in field situations. Although highly expressed, constitutive promoters are commonly used in proof-of-concept work, they may not always be the most suitable choice for development of commercial products. In previous work, we generated transgenic banana plants with TR4 resistance using the ZmUbi promoter driving *Ced*-9 (Paul et al., 2011) or the *A. tumefaciens* nopaline synthase (Nos) promoter driving *Mam*RGA2 (Dale et al., 2017a). Although Nos generally has low expression in bananas it was sufficient to confer field resistance against Foc TR4 using *Mam*RGA2. We have now identified several root-specific promoters with varying expression levels which will be suitable for developing TR4-resistant bananas using either *ced-9* or *Mam*RGA2. Further, combining the banana-derived TIP2 promoters described herein with the banana-derived *Mam*RGA2 will allow cisgenic constructs to be deployed for field resistance, avoiding the use of foreign sequences, and restricting expression to the roots. This may alleviate perceived concerns about expression of the resistance genes in the fruit of commercially grown Foc TR4-resistant transgenic banana plants.

## Data availability statement

The original contributions presented in the study are included in the article/**Supplementary Material**. Further inquiries can be directed to the corresponding author.

## Author contributions

JD, RH and AJ conceived and designed the research. AJ, J-YP, PD and JS performed the research. AJ, J-YP and TC analysed the data. AJ wrote the initial manuscript. J-YP, PD and RH revised the manuscript. JD and RH acquired funding and supervised the work. All authors read and approved the final version of the manuscript.

## Funding

This study received funding from an Australian Research Council Linkage Grant with financial support from LaManna Bananas Pty Ltd. The funder was not involved in the study design, collection, analysis, interpretation of data, the writing of this article or the decision to submit it for publication.

## Acknowledgments

The authors thank Ms Jen Kleidon for the provision and maintenance of banana ECS used in this study.

## Conflict of interest

The authors declare that the research was conducted in the absence of any commercial or financial relationships that could be construed as a potential conflict of interest.

## Publisher’s note

All claims expressed in this article are solely those of the authors and do not necessarily represent those of their affiliated organizations, or those of the publisher, the editors and the reviewers. Any product that may be evaluated in this article, or claim that may be made by its manufacturer, is not guaranteed or endorsed by the publisher.
