# Supplementary material for: Assessment of root-specific promoters in banana and tobacco and identification of a banana TIP2 promoter with strong root activity
Source: Front Plant Sci. 2022 Oct 5;13:1009487. doi: 10.3389/fpls.2022.1009487 (PMC9581176; doi:10.3389/fpls.2022.1009487)
Supplement: Supplementary file 1 [file Table_1.docx]

**Table S1**

List of primers used to amplify promoter sequences and for screening of cultures and transgenic plants

| Promoter target | Forward primer (5’🡪3’) | Reverse primer (5’🡪3’) | Amplicon size (bp) |
| --- | --- | --- | --- |
| AtRSK1a^1^ | GAAAATGAATCATTTGTGTTCAGA | TTTCAACTTCTTCTTTTGTGTTAT | 1197 |
| AtRSK1b | CCACCAGCTACAAAACCAATAAACCC |  | 2300 |
| AtCrp1 | GTAGTCCTTTCTCCTCATCCTCC | GCTCAGTTTCAGAGATTTGACGG | 2291 |
| AtCrp2 | GCTATGTATCACCCGGATGTGAAAC | CTGTTTGGATCTGGACCTGCGCG | 460 |
| AtEIR1a^1^ | TGTTGGTCAATTCACCGTTTTTTT | TTTGATTTACTTTTTCCGGCGAGA | 1218 |
| AtEIR1b | ATCATTACCAGTACCGAATGATGTG |  | 2157 |
| AtPyk10 | CTCGCCACTGCAACGAAGTGTACC | TTTGTAATTCTGATTTTATTCAAG | 1455 |
| BvMll | GTTTGTTAACTGAACTGAACTGTTAATT | TAAAATGGTTGGGCTAGGTGCCC | 1653 |
| BvTlp | CCCTCTATGCCTTCATAAACTGCC | CTTGGAAGTTTGAGTATTTTTTGGAG | 2090 |
| FaRB7 | GGATGACATGCATGTTGG | TTTGATCACTGGAAAAT | 974 |
| GmPRP1 | ACATCAATTTACTACAACT | CTCACTGTTAAGTGGTGC | 1041 |
| MaTIP2-2a | GTCATCTAGTGTACGAAGGCG | CAGAACAAGGACGAGTTACACC | 1483 |
| MaTIP2-2b | GACCTCAACCAATGGAGAG | GTTGTGTCACCGCTGGCCTCG | 801 |
| MsPRP2 | GATGCATGATTCGATTAC | GTTTCAAAGAAAGTAGTG | 649 |
| NtRB7a^1^ | TGAATTAGTTTGGTCATACGG | CTCACTAGAAAAATGCCCC | 684 |
| NtRB7b | GTTTCTTGCGTGTTGGAACGTC |  | 1321 |
| OsRCg2 | CTCAACAGTTTATTTTATATGATGG | TGCAGCTAGCGAGCTAGTGATCG | 1642 |
| ZmGLU1 | TCTTCTCAAGCTCACGGC | CCCCCTTTGCTAGCTAGC | 1720 |
| ZmPR10.1 | TGGTGGCCTGCCGACTCC | GGCTAGCTTAGCTTACTTGC | 1495 |

^1^ a denotes a 5’ truncated version of the same sequence described in b
