# Supplementary material for: Assessment of root-specific promoters in banana and tobacco and identification of a banana TIP2 promoter with strong root activity
Source: Front Plant Sci. 2022 Oct 5;13:1009487. doi: 10.3389/fpls.2022.1009487 (PMC9581176; doi:10.3389/fpls.2022.1009487)
Supplement: Supplementary file 2 [file Table_2.docx]

**Table S2**

*Musa* aquaporin sequences identified from the DH-Pahang assembly

| Aquaporin type | Identification | Chromosomal locus^a^ |
| --- | --- | --- |
| PIP1-1 | Aquaporin | Ma08_p11300.1 |
|  | Aquaporin | Ma04_p21270.1 |
| PIP1-2 | Probable aquaporin | Ma10_p07860.1 |
|  | Probable aquaporin | Ma05_p12170.1 |
|  | Probable aquaporin | Ma07_p18280.1 |
|  | Probable aquaporin | Ma02_p05980.1 |
|  | Probable aquaporin | Ma11_p07230.1 |
|  | Probable aquaporin | Ma05_p16880.1 |
|  | Probable aquaporin | Ma03_p14410.1 |
| PIP2-3 | Aquaporin | Ma08_p14890.1 |
|  | Aquaporin | Ma11_p00520.1 |
| PIP2-4 | Aquaporin | Ma04_p27400.1 |
|  | Aquaporin | Ma02_p00190.1 |
| PIP2-6 | Probable aquaporin | Ma02_p13080.1 |
|  | Probable aquaporin | Ma04_p22160.1 |
|  | Probable aquaporin | Ma01_p19750.1 |
|  | Probable aquaporin | Ma05_p11240.1 |
|  | Probable aquaporin | Ma08_p34310.1 |
| PIP2-7 | Aquaporin | Ma01_p19740.1 |
|  | Aquaporin | Ma01_p02770.1 |
| PIP-type | Aquaporin | Ma08_p34320.1 |
| TIP1-1 | Probable aquaporin | Ma08_p12690.1 |
|  | Probable aquaporin | Ma04_p20470.1 |
|  | Probable aquaporin | Ma11_p02140.1 |
|  | Probable aquaporin | Ma05_p21860.1 |
| TIP1-3 | Aquaporin | Ma10_p13630.1 |
|  | Aquaporin | Ma06_p16930.1 |
| TIP2-2 | Aquaporin | **Ma11_p04350.1** ^a^ |
|  | Aquaporin | **Ma11_p19380.1** ^a^ |
|  | Probable aquaporin | Ma06_p07460.1 |
|  | Probable aquaporin | Ma10_p22190.1 |
|  | Probable aquaporin | Ma09_p29530.1 |
| TIP3-2 | Aquaporin | Ma02_p12490.1 |
|  | Aquaporin | Ma04_p32570.1 |
| TIP4-3 | Aquaporin | Ma01_p07310.1 |
| TIP4-4 | Aquaporin | Ma10_p26220.1 |
|  | Aquaporin | Ma09_p06080.1 |
| TIP5-1 | Aquaporin | Ma04_p31110.1 |
| NIP1-1 | Aquaporin | Ma06_p16240.1 |
| NIP2-1 | Aquaporin | Ma06_p04340.1 |
|  | Aquaporin | Ma05_p25720.1 |
|  | Aquaporin | Ma10_p29710.1 |
|  | Aquaporin | Ma09_p28090.1 |
| NIP3-1 | Aquaporin | Ma04_p23930.1 |
|  | Aquaporin | Ma09_p19270.1 |
| NIP4-2 | Probable aquaporin | Ma09_p00700.1 |
| NIP5-1 | Probable aquaporin | Ma09_p19260.1 |
| SIP1-2 | Aquaporin | Ma11_p12650.1 |
| SIP2-1 | Aquaporin | Ma05_p29970.1 |
|  | Probable aquaporin | Ma05_p23920.1 |

^a^ Sequences in bold indicate those selected for this study.
