# Supplementary material for: Assessment of root-specific promoters in banana and tobacco and identification of a banana TIP2 promoter with strong root activity
Source: Front Plant Sci. 2022 Oct 5;13:1009487. doi: 10.3389/fpls.2022.1009487 (PMC9581176; doi:10.3389/fpls.2022.1009487)
Supplement: Supplementary file 3 [file Table_3.docx]

**Table S3**

Additional primers used for PCR screening work (together with primer Gus-R)

| Primer name | Primer sequence (5’ 🡪 3’) |
| --- | --- |
| Crp1-F | TCTCTCTTAAGAAGTCGGTG |
| EIR-F | TGCCGAGGAAGAAAAAAGGC |
| FaRB7-F | CCTTGGGTCCATCCTCTATG |
| Gus-R | CTAGAAATTTACCCTCAGATCTACC |
| Mll-F | ATAAGTGTACTGAGATAGTGTC |
| MusaTIP2a-F | GCTGTAAAGTGCACACCAACC |
| Pyk10-F | ACACGGACCGTGGTCTTTACC |
| RCg2-F | TGGTGAAGCTAGAGCTGTGC |
| RSK-F | ACTCAAAGGTCGGTCAGTACG |
| SbPRP1-F | CGCATATAGTACGTCGGTGC |
| Tlp-F | GATATTATGAGAAATGTCATACACG |
| TobRB7-F | TCATTTGTCCTAGTCCACTC |
| ZmGUS-F | AAGGCACTCGGCAAAGCTGC |
| ZmPR10-F | CATGGAAAAGACCGACGACTCG |
