# Supplementary material for: Assessment of root-specific promoters in banana and tobacco and identification of a banana TIP2 promoter with strong root activity
Source: Front Plant Sci. 2022 Oct 5;13:1009487. doi: 10.3389/fpls.2022.1009487 (PMC9581176; doi:10.3389/fpls.2022.1009487)
Supplement: Supplementary file 4 [file DataSheet_1.docx]

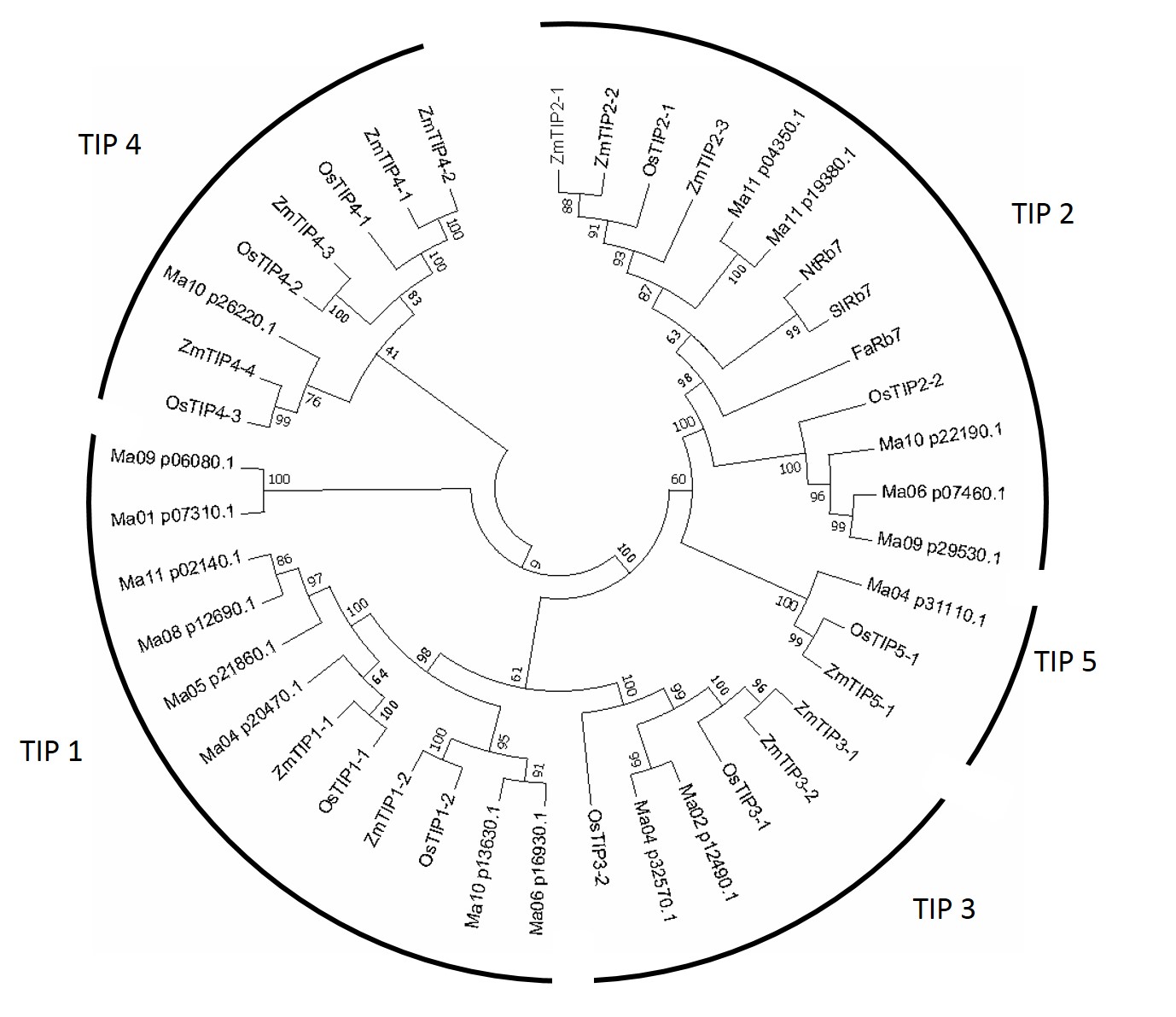


**Supplementary Figure 1.** Phylogenetic analysis of *Musa* tonoplast intrinsic protein (TIP) amino acid sequences. Putative *Musa acuminata* TIP amino acid sequences were identified in the DH Pahang v1 (released July 2012) genome sequence (<https://banana-genome-hub.southgreen.fr/>), downloaded and aligned together with rice (Sakurai et al., 2005) and maize (Chaumont et al., 2001) TIP sequences, as well as tobacco RB7 (NtRB7) (Yamamoto et al., 1991), strawberry RB7 (FaRB7) (Vaughan et al., 2006) and tomato RB7 (SlRB7) (GenBank accession no. AAB53329). Sequences were aligned in MEGA7 (Kumar et al., 2016) and phylogenetic reconstruction carried out using the Neighbor-Joining method with bootstrap analysis (1000 replications).
