# Supplementary material for: Assessment of root-specific promoters in banana and tobacco and identification of a banana TIP2 promoter with strong root activity
Source: Front Plant Sci. 2022 Oct 5;13:1009487. doi: 10.3389/fpls.2022.1009487 (PMC9581176; doi:10.3389/fpls.2022.1009487)
Supplement: Supplementary file 5 [file DataSheet_2.docx]

**
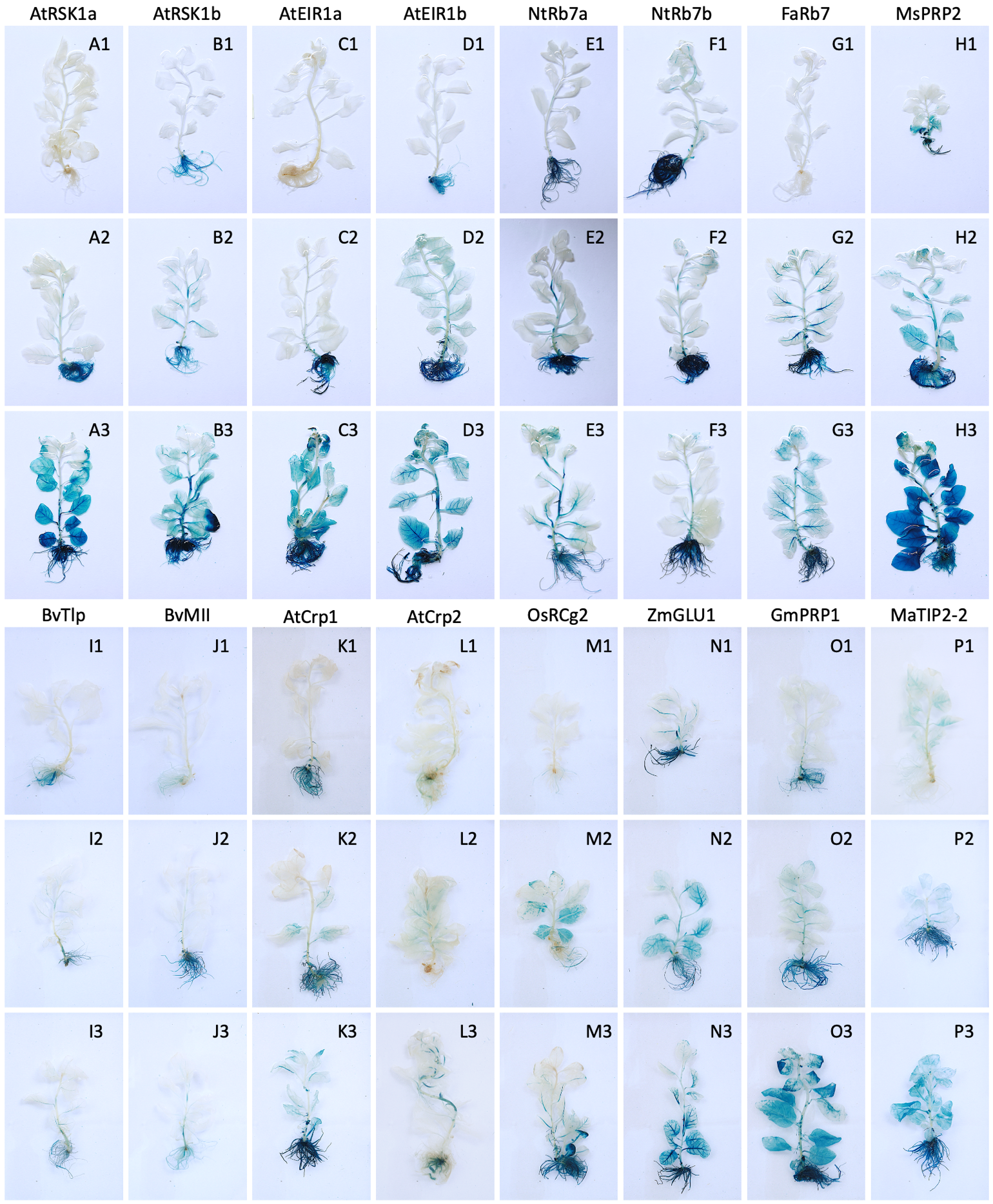
**

**Supplementary Figure 2.** Expression pattern of promoter-*uidA* constructs in whole plants of the transgenic tobacco lines. Transgenic promoter-*uidA* tobacco plants were generated and whole plants were stained to visualize GUS activity. Three plants are shown for each promoter-*uidA* construct. Promoter constructs shown include *Arabidopsis thaliana* root-specific kinase homolog 1a **(A)** and 1b **(B)**, *A. thaliana* ethylene insensitive root 1 a **(C)** and b **(D)**, *Nicotiana tobacum* Rb7a **(E)** and b **(F)**, *Fragaria ananassa* Rb7 **(G)**, *Medicago sativa* proline rich protein 2 **(H)**, *Beta vulgaris* thaumatin-like protein **(I)**, *B. vulgaris* major latex-like protein **(J)**, *A. thaliana* cryptic root-specific promoter 1 **(K)** and 2 **(L)**, *Oryza sativa* root-specific cDNA 2 **(M)**, *Zea mays* β-glucosidase 1 **(N)**, *Glycine max* proline-rich protein 1 **(O)** and *Musa acuminata* tonoplast intrinsic protein 2-2a **(P1)**, MaTIP2-2b **(P2 and P3)**.
